# Research on the Mechanism of Parent–Child Attachment to College Student Adversarial Growth

**DOI:** 10.3390/ijerph19073847

**Published:** 2022-03-24

**Authors:** Mi Tian, Ting Nie, Hengrui Liang

**Affiliations:** 1 School of Business, Macau University of Science and Technology, Macau 999078, China; mtian@must.edu.mo; 2 Shenzhen Xinbaoyou Industry Co., Ltd., Shenzhen 518020, China; henryliangt@gmail.com

**Keywords:** parent–child attachment, self-identity, adversarial growth, internal control personality, college student

## Abstract

This study explores the impact of parent–child attachment mechanisms on adversarial growth among Chinese students. After Chinese college students start independent life away from their parents, they face adversity on their own. However, their original family always influences students’ methods for dealing with adversity and how they grow and mature. A survey of 364 college students found that parental trust and communication have positive impacts on adversarial growth through the improvement of self-identity, while parental alienation reduces self-identity and contributes negative effects on the adversarial growth of college students. Internal control personality has a negative moderating effect between parental trust, parental communication, and adversarial growth and a positive moderating effect between parental alienation and adversarial growth. Low internal control personality therefore has a positive influence on parental trust and communication on adversarial growth and decreases the negative influence of parental alienation. A substitution effect between internal control personality and parental attachment was also found. Different child personality requires different type of parent–child attachment relationship to maximize their ability to handle future adversity.

## 1. Research background

During infancy and early childhood, individuals interact with their primary caregivers to form a stable cognition of their relationship, which subtly affects their behavioral and psychological development in adulthood [1]. The original family influence will therefore accompany an individual throughout their life, especially when responding to adversity and personal well-being [2,3]. Almost all people face adverse life experiences throughout their lives, but while some develop physical or psychological problems in response to adversity, others grow by acquiring new skills, rethinking their values, and exploring new opportunities through an experience of “adversarial growth” [4,5]. Is an individual’s original family dynamic a reason for their adversarial growth capabilities? Can a positive parent–child attachment relationship enhance an individual’s self-identity and help them cope positively with adversity? Finally, can an individual’s tendency to self-control facilitate this positive change?

In China, regulations for compulsory residence make almost all students leave their parents’ home when attending university, meaning that these students must confront difficult personal and academic changes independently [6]. At this transitional stage, an individual’s original family still has a deep imprint on their behavior, with each of their choices likely influenced by the parent–child attachment relationship. This study therefore analyzes the influence mechanism of parent–child attachment on Chinese college students’ adversarial growth while also accounting for the moderating effect of internal control personality.

## 2. Theoretical Basis and Research Hypothesis

Attachment is a close emotional connection between an infant and their early caregiver, which is formed at an early age in infants and young children that makes them seek support from attachment objects when they feel threatened or uncomfortable [1]. Initially, the definition of attachment was limited to explaining the emotional bond between mother and child, but with the enrichment of attachment theory, the notion of ‘attachment objects’ gradually expanded to include father and son and caregiver and child. Today, attachment relationships are considered to be the emotional connection between an individual and all attachment objects [7]. An individual’s early parent–child attachment affects their subsequent peer, and even adult, attachment relationships [8]. Armsden & Greenberg [9] have developed the Inventory of Parent and Peer Attachment (IPPA) to assess adolescent perceptions of positive and negative affective/cognitive relationships with their parents from three dimensions: parental trust (mutual understanding and respect, mutual trust), parental communication (extent and quality of verbal communication), and parental alienation (feelings of alienation and isolation). The assessment has verified the relationship between these attachment relationships and adolescent psychological well-being, while subsequent studies have also supported this conclusion. The secure attachment relationships formed in early childhood will have a positive impact on subsequent interpersonal communication, helping individuals make correct judgments and timely responses to others [10,11]. Active communication and mutual trust between parents and children helps children achieve better grades in school, better adapt to their environment and have higher perceptions of happiness [12,13]. In contrast, insecure attachment is related to internalization problems, anxiety or depression, and the triggering of negative emotions [14]. Alienated parent–child relationships may be induced by harmful adult behaviors such as addiction and gambling [15,16].

Tedeschi and Calhoun [17] believe that “post-traumatic growth” is a positive psychological change individuals exhibit following extremely challenging life events. Many people who have experienced trauma (including, but not limited to, the diagnosis of a chronic disease or terminal illness, loss of a loved one, experience of sexual assault, and natural disasters) not only demonstrate amazing mental resilience but also form a new understanding of life that allows them new development opportunities. Holman and Silver [5] argue that such adversity is multi-layered, with trauma as its highest degree but also, notably, uncommon: While trauma is not experienced by most people, adversity is faced by everyone. Joseph and Linley [18] maintain that “growth following adversity is about psychological well-being and changes in assumptions about the self and the world”. Coping with adversity results in higher levels of psychological functioning: people not only become more resilient, but use their adversity to further grow and develop [19]. At the same time, adversarial growth is different from resilience, in that, although it varies from person to person, it is not a personality trait but a complex process influenced by many contextual factors [18]. For many severely ill patients, partner support is a valid predictor of adversarial growth [20], while in the face of the severe COVID-19 epidemic, emotional support, information literacy, and religious coping have been found to induce adversarial growth [21]. There are multiple pressures that exceed expectations in the drastic changes that come with college life. Many of these students will be able to obtain adversarial growth by adjusting their coping strategies [6]. Allen [22] suggests that emotional acquisitiveness and parental response and encouragement are all conducive to the development of adolescent independence. In positive parent–child relationships, parents usually adopt a guiding and caring attitude towards their children’s growth, establishing mutual trust through frequent communication and effort rather than just setting rules. Parents create a relatively harmonious family atmosphere that allows their children to participate in family decision making, not only increasing the children’s skills but also giving them a stronger sense of self-identity. When these children encounter future adversity they will be more confident when dealing with and learning from difficulties. In negative parent–child relationships, the bond between parents and children is relatively alienated, with parents not paying enough attention to children’s needs. Consequently, when facing adversity, these children will be prone to negativity, lack self-confidence and minimize their chance for growth. Given this information, the present study proposes research Hypothesis 1:

**Hypothesis** **1a.** *Parental trust has a significant positive impact on their children’s adversarial growth.*

**Hypothesis** **1b.** *Parental communication has a significant positive impact on their children’s adversarial growth.*

**Hypothesis** **1c.** *Parental alienation has a significant negative impact on their children’s adversarial growth.*

Self-identity is a dynamic and continuous process in which an individual forms and develops through self-cognition. The process of identity development is relatively coordinated with the environment, that is, the individual’s relationship to the context they are currently facing. Self-identity is also formed through career choices, gender roles, and life values [23]. Overall, self-identity may be regarded as a kind of an individual’s internal identification or confirmation of their own role and a self-consciousness of this role based on self-experience as reflective understanding [24]. Because self-identity is a contextual process, Ahlquist [25] notes that an individual’s college experience influences the formation of their self-identity. The formation of individual self-identity is to a large extent a socialization process affected by personal relationships, defined by a person’s family role and social environment [26]. Positive parent–child attachment gives children enough safety and confidence to understand and explore the world [27], promoting individual self-identity by providing supportive autonomous communication [12]. Moreover, a high sense of self-identity can help individuals increase their confidence in coping with adversity, helping them land opportunities for growth while coping with adversity [28]. Parental trust and communication, hallmarks of a positive parent–child relationship, improve children’s self-evaluation, form a higher sense of self-identity, and face adversity more calmly. In contrast, parental alienation makes individuals feel neglected and despised, which may prevent the formation of individual self-identity and is even more detrimental to growth in adversity. Therefore, the present study proposes research Hypothesis 2:

**Hypothesis** **2a.** *Parental trust significantly affects their children’s adversarial growth through self-identity.*

**Hypothesis** **2b.** *Parental communication significantly affects their children’s adversarial growth through self-identity.*

**Hypothesis** **2c.** *Parental alienation significantly affects their children’s adversarial growth through self-identity.*

While both internal and external control personalities generally believe that behavior reinforces consequences, internal control personality individuals attribute their successes or failures to their own efforts [29]. These individuals firmly believe that personal factors such as ability, knowledge, and skills are decisive in affecting work and life. Consequently, the internal control personality is a popular topic in personality research. Internal and external control personality traits have been found to effectively explain differences in individual work behaviors [30], with internal control personality individuals demonstrating greater loyalty to their employer organization and more innovative performance strategies, resulting in stronger job satisfaction [31]. Internal control personality individuals tend to actively face obstacles and challenges, are good at learning, and find effective solutions to solving existing problems [32,33,34]. An internal control personality therefore has a positive impact on corporate innovation and individual risk-taking, contributing to corporate innovation and entrepreneurial success [35]. Both social support and family support can help individuals with internal control personality achieve academic and entrepreneurial success [36,37]. As a positive personality trait, the internal control personality allows individuals to handle difficulties more effectively and should thus enhance positive parent–child relationships to promote the adversarial growth, as well as restrain negative parent–child relationships that hinder this growth. The present research proposes Hypothesis 3:

**Hypothesis** **3a.** *Internal control personality has a positive moderating effect on the relationship between parental trust and their children’s adversarial growth, and high internal control personality can enhance the positive impact of parental trust on their children’s adversarial growth.*

**Hypothesis** **3b.** *Internal control personality has a positive moderating effect on the relationship between parental communication and their children’s adversarial growth, and high internal control personality can enhance the positive impact of parental communication on their children’s adversarial growth.*

**Hypothesis** **3c.** *Internal control personality has a negative moderating effect on the relationship between parental alienation and their children’s adversarial growth. High internal control personality can weaken the negative impact of parental alienation on their children’s adversarial growth.*

## 3. Research Design

### 3.1. Procedure

In the Chinese education system, university is the beginning of a student’s individual independence. In primary and secondary schools, students go home after school and live with their parents, but upon entering university, barring special exceptions, all students must live on their university campus, even if they are local students. Therefore, almost all Chinese students at the university level are confronted with the difficulties of both studying and living independently. Just beyond their parents’ protective reach, Chinese students’ behaviors are likely to show traces of their parents’. Therefore, the present study examined a sample of Chinese college students to explore the enduring influence of the parent–child attachment on students’ individual adversarial growth. We conducted statistical surveys on students in Beijing, Shanghai, Macau, Guangdong, Guangxi, Sichuan, Yunnan, Guizhou, Fujian, and other provinces, municipalities, and autonomous regions at the beginning of 2020. A total of 500 paper and online questionnaires were distributed through convenient sampling. The survey was conducted on a voluntary basis, and 109 respondents refused to participate. Participants can choose a paper questionnaire or an online questionnaire. A total of 391 questionnaires were returned (86 paper questionnaires and 305 online questionnaires), with a response rate of 78.2% and 364 valid questionnaires (85 paper questionnaires and 279 online questionnaires), creating an effective response rate of 72.8%. All respondents were students living on university campuses, with 151 under the age of 20 (41.5% of the total sample), 184 respondents between 20 and 30 years old (50.6%), and only 29 respondents over 30 (8%). These numbers basically align with the age distribution of Chinese college students. There were 190 male respondents (52.2% of the total sample) and 174 female respondents (47.8%), meaning a relatively balanced male-to-female ratio, reflecting the overall gender ratio in the Chinese higher education system. The vast majority of respondents were undergraduate students, with 240 undergraduate students accounting for 65.9% of the total sample, and 124 graduate students accounting for 34.1% of the total sample. This reflects a relatively high rate of graduate student representation when compared with the ratio of undergraduates to postgraduates in China’s higher education system. Respondent majors included business, engineering, liberal arts, science, architecture, and others, covering the main majors set up by Chinese universities.

### 3.2. Measurement

The scales used in this study refer to the English and Chinese versions of the scales in previous studies. Standard and back-translation procedures were used to ensure scale accuracy and completeness.The reliability and validity of the scale were verified by pre-testing. The questionnaire included descriptions of the research purpose, an academic research confidentiality statement, demographic variables, parental attachment, self-identity, adversarial growth, and internal control personality, which were measured via Likert five-point scales from 1—completely disagree to 5—completely agree. The demographic variables involved in the survey mainly include gender (Male;Female), age (Less than 20; 20–29; More than 30), education (Technical College Student; University Undergraduate; Postgraduate; PhD student), and major (Management; Economics; Law; Education; Literature; History; Science;Engineering; Agriculture; Medicine; Philosophy; Art; Other). Respondents were told to answer voluntarily and could stop at any time. Incomplete questionnaires were counted as invalid. Data were analyzed by SPSS 24, Amos24, and Process V3.4 statistical software and mainly related to descriptive statistics, confirmatory factor analysis, correlation analysis, hierarchical regression analysis, and bootstrap analysis to validate the study’s research hypotheses.

Parent–child attachment refers to adolescents’ perceptions of the positive and negative affective/cognitive relationships with their parents, which has three dimensions: parental communication (extent and quality of verbal communication), parental trust (mutual understanding and respect, mutual trust), and parental alienation (feelings of alienation and isolation) [9]. The measurement of parent–child attachment is based on the Inventory of Parent and Peer Attachment (IPPA) [9], which contains 25 items in 3 dimensions, and the internal consistency coefficients of parental trust, parental communication, parental alienation are 0.876 (parental communication), 0.847 (parental trust), and 0.794 (parental alienation), respectively. Self-identity refers to an individual’s confirmation of one’s own role and a self-consciousness formed by an individual based on self-experience as a reflective understanding [24]. The measurement of self-identity is based on the Self-Identity Scale(SIS) [38]. The scale contains 19 items. The internal consistency coefficient is 0.879. Adversarial growth refers to psychological well-being and changes in assumptions about the self and the world after adversity [18]. The measurement of adversarial growth is based on the short form of the Post-traumatic Growth Inventory (PTGI-SF) [39], which has 10 items and an internal consistency coefficient of 0.840. Internal control personality individuals attribute their successes or failures to their own efforts (Rotter, 1966). Measurement of internal control personality is based on the Work Locus of Control Scale (WLCS) [40]. The scale contains 16 items with an internal consistency coefficient of 0.902.

### 3.3. Correlation Analysis

Through correlation analysis, we have a preliminary understanding of the relationship between the various variables in this study. After controlling gender, age, education and major, parent–child attachment, self-identity, adversarial growth, and self-control personality have significant correlations. Parental trust and parental communication has a significant positive correlation with individual’s self-identity (0.246 **, 0.422 **) and adversarial growth (0.282 **, 0.348 **). The higher the degree of parental trust and communication are, the stronger the individual’s sense of self-identity is. When encountering adversity, they will show a greater possibility of growth. Parental alienation has a significant negative correlation with the individual’s self-identity (−0.265 **) and adversarial growth (−0.255 **). The more alienated the relationship between a child and their parents is in the original family, the lower their sense of self-identity will be as an adult. Their adversarial growth level is also lower. Self-identity and individual adversarial growth have a significant positive correlation (0.641 **). Individuals with lower self-identity also have lower levels of adversarial growth. Self-control personality has a weak correlation with self-identity (0.282 **) and adversarial growth (0.220 **).

## 4. Results

### 4.1. Descriptive Statistics

Through the descriptive statistical analysis, we can come to know the perceived level of parent–child attachment, self-identity, adversarial growth, and internal control personality in this survey, with the results shown in Table 1. Most individuals believe that they still have a trusting relationship with their parents (3.54), but the communication with to parents is weakening (3.24). The parental alienation is not serious (3.10), indicating that most of the respondents have a positive parent–child attachment relations, and their self-identity is at a medium-to-high level, which means that they have always held a positive attitude towards themselves (3.79). When confronted with adversity, the survey respondents show a tendency to grow at a moderate level. They would rethink their goals and life direction in the face of adversity (3.64). The internal control personality of the respondents in this survey is relatively obvious (3.79), which has a certain relationship with the modern educational philosophy and the characteristics of the times.

Through correlation analysis, we have a preliminary understanding of the relationship between the various variables in this study. After controlling for gender, age, education and major, parent–child attachment, self-identity, adversarial growth, and self-control personality have significant correlations. Parental trust and parental communication can significantly predict the individual’s self-identity (0.246 **, 0.422 **) and growth in adversity (0.282 **, 0.348 **). The higher the degree of parental trust and communication are, the stronger the individual’s sense of self-identity is. When encountering adversity, they will show the greater possibility of growth. Parental alienation has a significant negative impact on the individual’s self-identity (−0.265 **) and adversarial growth (−0.255 **). The more alienated the relationship between a child and their parents is in the original family, the lower their sense of self-identity will be as an adult, which is also not conducive to the occurrence of adversarial growth. Self-identity and adversarial growth have a significant positive impact (0.641 **), and self-identity can promote the growth of individuals under adversity. Self-control personality has a weak correlation with self-identity (0.282 **) and adversarial growth (0.220 **).

### 4.2. Regression Analysis

**Figure 1:**
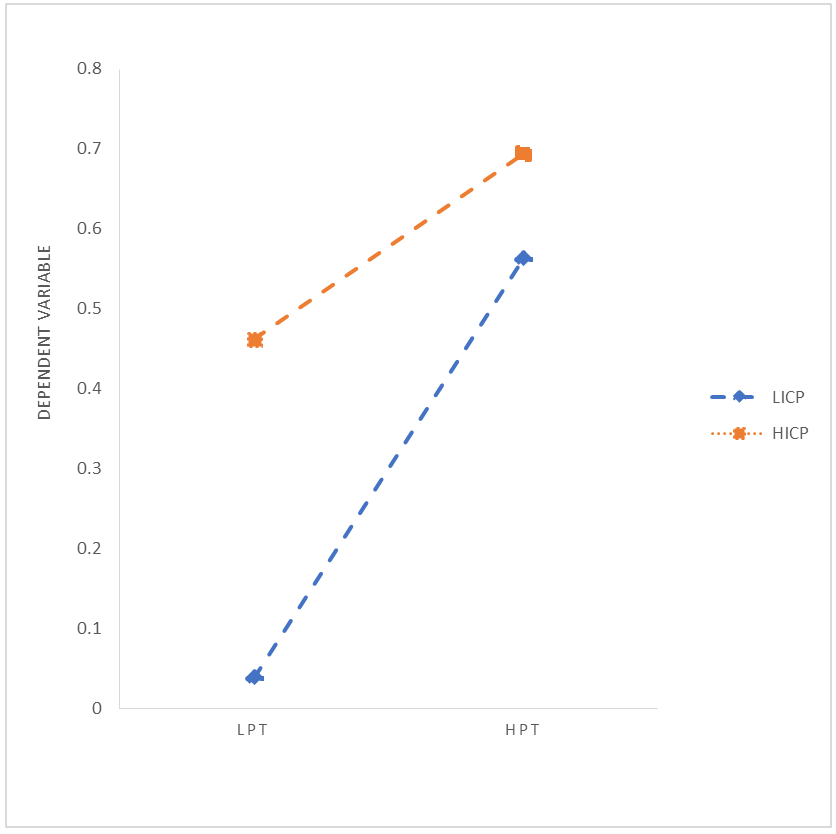
Moderating Effects of Internal Control Personality (Parental Trust). Notes: LPT: Low Parental Trust, HPT: High Parental Trust; LPC: Low Parental Communication, HPC: High Parental Communication; LPA: Low Parental Alienation, HPA: High Parental Alienation; LICP: Low Internal Control Personality, HICP: High Internal Control Personality.

**Figure 2:**
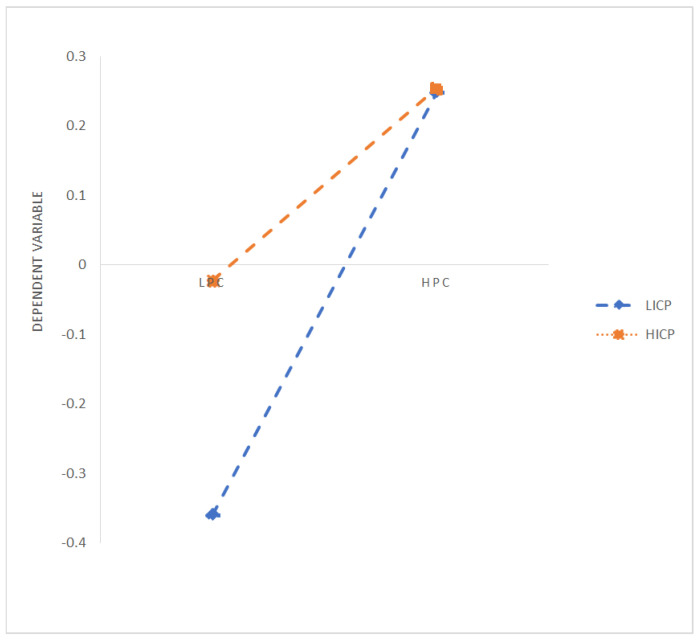
Moderating Effects of Internal Control Personality (Parental Communication). Notes: LPT: Low Parental Trust, HPT: High Parental Trust; LPC: Low Parental Communication, HPC: High Parental Communication; LPA: Low Parental Alienation, HPA: High Parental Alienation; LICP: Low Internal Control Personality, HICP: High Internal Control Personality.

**Figure 3:**
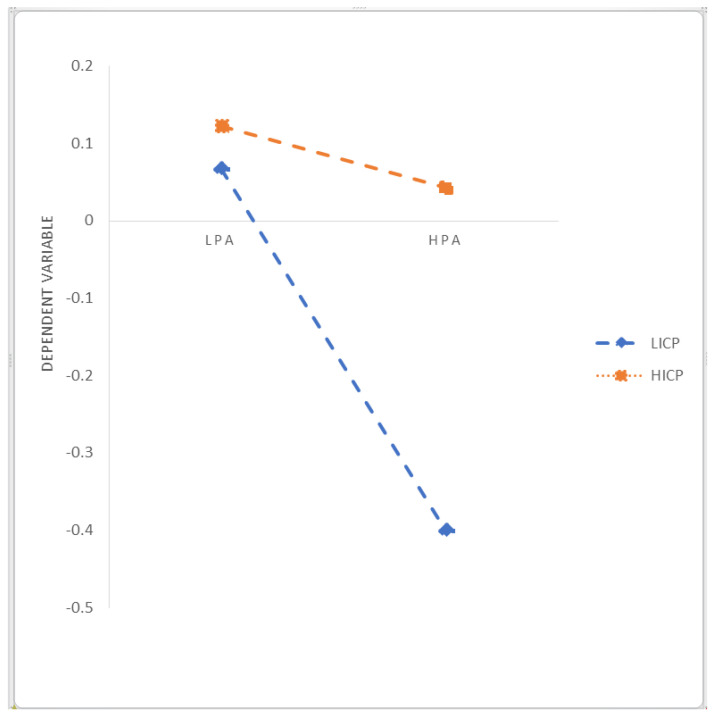
Moderating Effects of Internal Control Personality (Parental Alienation). Notes: LPT: Low Parental Trust, HPT: High Parental Trust; LPC: Low Parental Communication, HPC: High Parental Communication; LPA: Low Parental Alienation, HPA: High Parental Alienation; LICP: Low Internal Control Personality, HICP: High Internal Control Personality.

Hierarchical regression is used to verify the influence of parent–child attachment on individual self-identity and adversarial growth, as well as the moderating role of internal control personality in parent–child attachment and adversarial growth. The specific analysis results are shown in Table 2. Models 1 to 3 verify the influence of parent–child attachment on self-identity. After controlling for gender, age, and education and major, parental trust (0.258 ***) and parental communication (0.440 ***) positively affect self-identity, and parental alienation (−0.260 ***) significantly negatively affects individual self-identity. Models 4 to 6 verify the influence of the parent–child attachment on adversarial growth. After controlling for gender, age, and education and major, parental trust (0.283 ***) and parental communication (0.357 ***) positively affect adversarial growth, and parental alienation (−0.258 ***) significantly negatively affects individual adversarial growth. Therefore, research Hypotheses 1a–c are verified. Model 7 verifies the influence of self-identity on adversarial growth. After controlling for gender, age, education, and major, self-identity positively affects adversarial growth (0.643 ***). Models 8 to 10 examine the moderating role of internal control personality on the relationship between parent–child attachment and adversarial growth. Model 8 explores the moderating effect of internal control personality on the relationship between parental trust and adversarial growth. After centralizing the related variables, parental trust, internal control personality, and interaction terms are brought into the regression equation with adversarial growth as the dependent variable. Parental trust (0.362 ***), internal control personality (0.328 ***), and the interaction term (−0.226 ***) have significant influence, so internal control personality has a negative moderating effect between parental trust and adversarial growth. Internal control personality will weaken the influence of parental trust on adversarial growth. It is inconsistent with the expected research Hypothesis 3a, so research Hypothesis 3a has not been verified. Model 9 examines the moderating effect of internal control personality in the relationship between parental communication and adversarial growth. After centralizing the relevant variables, the parental communication, internal control personality, and interaction term are brought into the regression equation with adversarial growth as the dependent variable. Parental communication (0.353 ***), internal control personality (0.203 ***), and interaction terms (−0.240 ***) have significant influences, so internal control personality has a negative moderating effect between parental communication and adversarial growth. Internal control personality will weaken the influence of parental communication on adversarial growth. It is inconsistent with the expected research Hypothesis 3b, so research Hypothesis 3b has not been verified. Model 10 examines the moderating effect of internal control personality in the relationship between parental alienation and adversarial growth. After centralizing the relevant variables, parental alienation, internal control personality, and the interaction term are brought into the regression equation with adversarial growth as the dependent variable. Parental alienation (−0.296 ***), internal control personality (0.295 ***), and interaction terms (0.291 ***) have significant influences, so internal control personality has a positive moderating effect between parental alienation and adversarial growth. Internal control personality will strengthen the influence of parental alienation on adversarial growth. This is inconsistent with the expected research Hypothesis 3c, so research Hypothesis 3c has not been verified. The specific moderating diagram is shown in Figure 1 (Parental Trust), Figure 2 (Parental Communication), and Figure 3 (Parental Alienation).

### 4.3. Mediating Effect and Moderating Effect

The process procedure is used to verify the mediating effect of individual self-identity between parent–child attachment and adversarial growth, and the moderating effect of internal control personality between parent–child attachment and adversarial growth at different levels. The results are shown in Table 3:

First of all, we explore the mediating effect of individual self-identity between parent–child attachment and adversarial growth. The parent–child attachment will be discussed into three types: parental trust, parental communication, and parental alienation. The 95% confidence interval of the direct effect of self-identity between parental trust and adversarial growth (b = 0.1128, SE = 0.0305) is (0.0528, 0.1729), and the interval does not pass zero; the 95% confidence interval of the indirect effect of self-identity between parental trust and adversarial growth (b = 0.0974, SE = 0.0231) is (0.0532, 0.1446), and the interval does not pass zero, which means self-identity has a mediating effect between parental trust and adversarial growth. The 95% confidence interval of the direct effect of self-identity between parental communication and adversarial growth (b = 0.0763, SE = 0.0373) is (0.0031, 0.1496), and the interval does not pass zero; the 95% confidence interval of the indirect effect of self-identity between parental communication and adversarial growth (b = 0.2011, SE = 0.0337) is (0.1388, 0.2717), and the interval does not pass zero, which means self-identity has a mediating effect between parental communication and adversarial growth. The 95% confidence interval of the direct effect of self-identity between parental alienation and adversarial growth (b = −0.0758, SE = 0.0328) is (−0.3085, −0.1229), and the interval does not pass zero; the 95% confidence interval of the indirect effect of self-identity between parental alienation and adversarial growth (b = −0.1147, SE = 0.0276) is (−0.1727, −0.0633), and the interval does not pass zero, which means self-identity has a mediating effect between parental alienation and adversarial growth. Therefore, research Hypotheses 2a–c have been verified.

Then, we explore the moderating effect of internal control personality between parent–child attachment and adversarial growth. Three types of the parent–child attachment relationship will also be discussed: parental trust, parental communication, and parental alienation. At a low standard of internal control personality, the 95% confidence interval of the moderating effect between parental trust and adversarial growth (b = 0.2053, SE = 0.0451) is (0.1166, 0.2939), and the interval does not pass zero, which means internal control personality at a low level has a moderating effect between parental trust and adversarial growth, namely, a low level of internal control personality can strengthen the positive influence of parental trust on adversarial growth. Meanwhile, at a high level of internal control personality, the 95% confidence interval of the moderating effect between parental trust and adversarial growth (b = 0.0435, SE = 0.0410) is (−0.0371, 0.1241), and the interval does pass zero, which means internal control personality at a high level does not have moderating effect between parental trust and adversarial growth. At a low standard of internal control personality, the 95% confidence interval of the moderating effect between parental communication and adversarial growth (b = 0.2214, SE = 0.0502) is (0.1226, 0.3202), and the interval does not pass zero, which means internal control personality at a low level has a moderating effect between parental communication and adversarial growth, namely, a low level of internal control personality can strengthen the positive influence of parental communication on adversarial growth. Meanwhile, at a high level of internal control personality, the 95% confidence interval of the moderating effect between parental communication and adversarial growth (b = −0.0324, SE = 0.0476) is (−0.1261, 0.0612), and the interval does pass zero, which means internal control personality at a high level does not have a moderating effect between parental communication and adversarial growth. At a low standard of internal control personality, the 95% confidence interval of the moderating effect between parental alienation and adversarial growth (b = −0.2157, SE = 0.0472) is (−0.3085, −0.1229), and the interval does not pass zero, which means internal control personality at a low level has a moderating effect between parental alienation and adversarial growth, namely, a low level of internal control personality can weaken the negative influence of parental alienation on adversarial growth. Meanwhile, at a high level of internal control personality, the 95% confidence interval of the moderating effect between parental alienation and adversarial growth (b = 0.0291, SE = 0.0440) is (−0.0574, 0.1155), and the interval does pass zero, which means internal control personality at a high level does not have a moderating effect between parental alienation and adversarial growth.

## 5. Conclusions and Discussion

Through the data analysis, it is verified that parent–child attachment has an effect on adversarial growth through self-identity. When an individual has a trust and communication relationship with their parents, it is easier for them to form a sense of self-identity. When facing adversity in life, it is more likely to make positive changes and promote one’s own development. However, when an individual is in an alienated relationship with his parents, it is not conducive to the formation of self-identity. When encountering difficulties, they are more likely to have negative emotions, which will adversely affect their own mental health. However, in this study, the moderating effect of internal control personality is inconsistent with the expected direction. Statistical analysis shows that the internal control personality plays a negative role in the relationship between parental trust and parental communication on adversarial growth. Low internal control personality will strengthen the promotion of parental trust and parental communication on adversarial growth. Internal control personality plays a positive role in the relationship between parental alienation on adversarial growth. A low internal control personality will decrease the negative influence of parental alienation on adversarial growth. When individuals have a low level of internal control, they will rely more on parent–child attachment.They will be more concerned with their relationship with their parents and will try to seek self-identity from attachment. The participants in this survey are all Chinese college students. The university stage is the beginning of their independent life, and their mindset and behaviors are greatly influenced by parent–child attachment in their original family. Parent–child attachment and internal control personality have a substitution effect. Individuals with high internal control personality are more confident in their own abilities, and they will try to solve problems by themselves, even in the face of adversity. Individuals with low internal control are more dependent on their parents, hoping to receive their parents’ advice and help. Positive parent–child attachment is more important to them, and their parents’ support helps them gain the confidence to overcome difficulties. When individuals are in an alienated parent–child relationship, it is difficult for them to obtain the approval and support from their original family; therefore many individuals with low internal control personality may give up their expectations on the family. They should rely more on their own efforts.

There are a few research limitations regarding sample age generalization and questionnaire-matching, impact bias, and non-response bias. All survey participants are college students with a high age concentration, therefore, such a conclusion does not necessarily generalize to all age groups. The external validity of this study may thus be affected. The Inventory of Parent and Peer Attachment (IPPA) is an effective tool for measuring adolescents, and Chinese college students generally start their freshman life during the last year of adolescence (19 years old). The effectiveness and biases caused by such difference require further examinations. Although the homology bias is not serious through statistical analysis, such personal perception variables based on self-rating scales still affect the research results comparing with paired survey. In this voluntary survey, participants may reject participation at any stage of the survey, and researchers remain unaware of their basic information or the reason why they rejected answering. Questionnaires are delivered softcopies (online) and hardcopies parallel to each other; hence, this simultaneous usage may lead to biasing effects.

Social development has led to intensified competition in the labor market, and the younger generations are facing growing challenges. They will inevitably experience all kinds of adversities as they grow up. Being capable of facing adversity calmly and learning to grow from it is a necessary element for future success. In the past 20 years, a large number of studies have demonstrated that individuals may experience positive changes following trauma [17], which focus on individuals who have experienced major life traumas, such as widowhood, fatal illness, earthquakes, rape, etc. Holman, & Silver [5] pointed out that trauma is the highest level of adversity and is not universal. Everyone faces different degrees of adversity, and also grows from adversity [18]. At present, there are relatively few empirical studies on adversarial growth. In addition, existing studies mainly explain the occurrence of adversity growth from the perspective social support and coping styles [6,21]. Few studies have discussed from a developmental perspective whether the original family may have an impact on the individual’s growth in adversity. Numerous studies have confirmed the importance of parent–child attachment at an early age to individuals’ physical and mental health in adulthood [3]. Therefore, we also believe that positive parent–child attachment can help individuals gain self-identity and increase the possibility of their adversarial growth, especially for individuals with low self-control personality, this promotion effect is more obvious.

China’s education system is relatively special. In primary and secondary schools, almost all students study in their local area and live with their parents. At the university level, school choices are no longer geographically restricted, but all students are required to live on campus and begin living independently. The independence of most Chinese children is not a gradual process, but is mandatory due to entering university. The statistical survey of Chinese college students basically verified our research hypothesis. The parent–child attachment relationship of the original family does have an impact on individual adversarial growth, and has a certain substitution effect with the internal control personality. The parent–child attachment relationship is not only very important in childhood, but its impact will continue into adulthood, which directly affects the individual’s coping style in the face of adversity. Parents need to work on forming a positive parent–child relationship, giving them more trust and support, and making them more confident in dealing with the uncertainties in the growing process. Especially for individuals with low internal control personalities, their expectations and dependence on positive family relationships are stronger, and they are more likely to benefit from positive parent–child attachment. The alienated parent–child relationship may cause the individual to fall into a hopeless state, which will seriously affect their physical and mental health and future development. Of course, whether an individual can grow under adversity is a very complicated process: It is not only affected by the original family but also by the growing environment, peer interaction, acquired learning, and other factors, which are worthy of further research and verification.

## Figures and Tables

**Table 1:** Means, Standard Deviations, and Correlations (N = 364).

	Mean	SD	1	2	3	4	5	6	7	8	9
Gender	1.55	0.499									
Age	2.04	1.068	−0.100								
Education	2.07	0.639	0.020	−0.202 **							
Major	4.51	3.167	0.071	−0.125 *	−0.050						
PT	3.54	1.225	0.119 *	0.084	−0.014	0.032					
PC	3.24	1.027	0.063	−0.051	0.056	0.042	0.537 **				
PA	3.10	1.120	0.090	0.020	−0.019	−0.125 *	−0.636 **	−0.476 **			
SI	3.79	0.774	−0.118 *	0.082	−0.030	0.017	0.246 **	0.422 **	−0.265 **		
AG	3.64	0.885	−0.030	0.086	0.004	0.014	0.282 **	0.348 **	−0.255 **	0.641 **	
IC	3.79	1.045	−0.014	0.064	0.013	−0.032	−0.221 **	0.024	0.138 **	0.282 **	0.220 **

Note: N = 364; ** *p* < 0.01, * *p* < 0.05; PT: Parental Trust, PC: Parental Communication, PA: Parental Alienation, SI: Self-Identity, AG: Adversarial Growth, IC: Internal Control Personality.

**Table 2:** Hierarchical Regression Results (N = 364).

	M1	M 2	M 3	M 4	M 5	M 6	M 7	M8	M 9	M10
	SI	SI	SI	AG	AG	AG	AG	AG	AG	AG
Gender	−0.146 *	−0.136 *	−0.087	−0.059	−0.042	0.003	0.049	0.075	−0.040	−0.006
Age	0.050	0.060	0.069	0.069	0.088	0.093	0.054	0.013	0.097	0.088
Education	−0.013	−0.034	−0.018	0.023	0.007	0.018	0.032	−0.031	−0.007	0.020
Major	0.023	0.021	0.000	0.017	0.017	−0.006	0.006		0.022	−0.016
PT	0.258 ***			0.283 ***				0.362 ***		
PC		0.440 ***			0.357 ***				0.353 ***	
PA			−0.260 ***			−0.258 ***				−0.296 ***
SI							0.643 ***			
IC								0.328 ***	0.203 ***	0.295 ***
PA1*IC								−0.226 ***		
PA2*IC									−0.240 ***	
PA3*IC										0.291 ***
$\Delta R^{2}$	0.085	0.219	0.086	0.088	0.134	0.074	0.415	0.220	0.233	0.220
F	5.676 ***	16.134 ***	5.696 ***	5.845 ***	9.426 ***	4.836 ***	43.170 ***	12.763 ***	13.805 ***	12.796 ***

Note: N = 364; *** *p* < 0.001, * *p* < 0.05; PT: Parental Trust, PC: Parental Communication, PA: Parental Alienation, SI: Self-Identity, AG: Adversarial Growth, IC: Internal Control Personality.

**Table 3:** Mediating Effect and Moderating Effect (N = 364).

Variable PT	Mediating Effect					Moderating Effect				
		Effect	SE	95% confidence interval			Effect	SE	95% confidence interval	
				Lower	Upper				Lower	Upper
	DE	0.1128	0.0305	0.0528	0.1729	L	0.2053	0.0451	0.1166	0.2939
	IDE	0.0974	0.0231	0.0532	0.1446	H	0.0435	0.0410	−0.0371	0.1241
Variable PC	Mediating Effect					Moderating Effect				
		Effect	SE	95% confidence interval			Effect	SE	95% confidence interval	
				Lower	Upper				Lower	Upper
	DE	0.0763	0.0373	0.0031	0.1496	L	0.2214	0.0502	0.1226	0.3202
	IDE	0.2011	0.0337	0.1388	0.2717	H	−0.0324	0.0476	−0.1261	0.0612
Variable PA	Mediating Effect					Moderating Effect				
		Effect	SE	95% confidence interval			Effect	SE	95% confidence interval	
				Lower	Upper				Lower	Upper
	DE	−0.0758	0.0328	−0.3085	−0.1229	L	−0.2157	0.0472	−0.3085	−0.1229
	IDE	−0.1147	0.0276	−0.1727	−0.0633	H	0.0291	0.0440	−0.0574	0.1155

Note: PT: Parental Trust, PC: Parental Communication, PA: Parental Alienation, SI: Self-Identity, AG: Adversarial Growth, IC: Internal Control Personality.

## Data Availability

The data presented in this study are available on request from the corresponding author.

## Abbreviations

The following abbreviations are used in this manuscript:

PT	Parental Trust
PC	Parental Communication
PA	Parental Alienation
SI	Self-identity
AG	Adversarial Growth
IC	Self-control personality
